# Supplementary material for: Synthetic control over the binding configuration of luminescent sp3-defects in single-walled carbon nanotubes
Source: Nat Commun. 2021 Apr 9;12:2119. doi: 10.1038/s41467-021-22307-9 (PMC8035247; doi:10.1038/s41467-021-22307-9)
Supplement: Supplementary file 1 — Supplementary Information [file 41467_2021_22307_MOESM1_ESM.pdf]

SUPPLEMENTARY INFORMATION

Synthetic control over the binding configuration of luminescent sp^3-defects in single-walled carbon nanotubes

Simon Settele[1], Felix J. Berger[1,2], Sebastian Lindenthal[1], Shen Zhao[3], Abdurrahman Ali El Yumin[1,2], Nicolas F. Zorn,[1,2], Andika Asyuda[1], Michael Zharnikov[1], Alexander Högele[3,4] and Jana Zaumseil[1,2]*

[1] Institute for Physical Chemistry, Universität Heidelberg, D-69120 Heidelberg, Germany

[2] Centre for Advanced Materials, Universität Heidelberg, D-69120 Heidelberg, Germany

[3] Fakultät für Physik, Munich Quantum Center, and Center for NanoScience (CeNS), Ludwig-Maximilians-Universität München, D-80539 München, Germany

[4] Munich Center for Quantum Science and Technology (MCQST), D-80799 München, Germany

* corresponding author: *zaumseil@uni-heidelberg.de*

CONTENTS

Supplementary Methods 1 3

Raman Spectra of Functionalized (6,5) SWNTs 3

Fluorescence Lifetime Measurements 3

Normalization of Photoluminescence Spectra 3

Photoluminescence Quantum Yield Measurements 4

Low and Variable Temperature Spectroscopy of SWNTs 5

Room Temperature Spectroscopy of Individual SWNTs and Autocorrelation Measurements 5

Detailed sp^3 Functionalization Protocol 6

Supplementary Table 1 | Example Values for Reaction Mixture 8

Supplementary Figure 1| Spectroscopic Characterization of PFO-BPy Wrapped (6,5) SWNTs 9

Supplementary Note 1: Concentration Effects 10

Supplementary Figure 2 | Concentration Effects on the Functionalization Process 10

Supplementary Figure 3 | Functionalization with Different Aniline Derivatives 11

Supplementary Figure 4 | Raman Spectra of Functionalized (6,5) SWNTs 11

Supplementary Figure 5 | Power-Dependence of $E_{11}*^-$ Defect State Photoluminescence 12

Supplementary Figure 6 | Defect State Power-Dependence Depending on Optical Trap Depth 13

Supplementary Figure 7 | Defect State Photoluminescence Decay Time Traces 14

Supplementary Table 2 | Overview of Different Defect State Photoluminescence Lifetimes 14

Supplementary Note 2: Temperature Dependence of Defect State Photoluminescence 15

Supplementary Figure 8 | Temperature Dependent Defect State PL in Thin Films 17

Supplementary Figure 9 | Effect of Optical Trap Depth on Temperature Dependent Defect PL (Film) 18

Supplementary Figure 10 | Temperature Dependent Defect State PL in SWNT Dispersion 19

Supplementary Figure 11 | Effect of Optical Trap Depth on Temperature Dependent Defect PL (Dispersion) 19

Supplementary Figure 12 | Cumulative PL Spectrum from Individual (6,5) SWNTs 20

Supplementary Figure 13 | Absorption Characteristics of Polymer Wrapped Large Diameter SWNTs 21

Supplementary Figure 14 | Characterization of (7,5) SWNTs Functionalized with 2-Iodoaniline 22

Supplementary Figure 15 | Diameter-Dependent Defect Properties of Functionalized SWNTs 23

Supplementary Note 3: Mechanistic Considerations 24

Supplementary Figure 16 | XPS of (6,5) SWNTs Functionalized with 5-Fluoro-2-iodoaniline 27

Supplementary Table 3 | Reference Experiments with (6,5) SWNTs in Toluene 28

Supplementary Table 4 | Reference Experiments with (6,5) SWNTs in THF 31

Supplementary Note 4: Non-Aniline Reagents 34

Supplementary Figure 17 | Functionalization with Non-Aniline Reagents 34

Supplementary Table 5 | $E_{11}*^-$ Optical Trap Depths of Functionalized (6,5) SWNTs 35

Supplementary Note 5: Impact of Oxygen/Water on Functionalization 36

Supplementary Figure 18 | Functionalization under Atmospheric Conditions 36

Supplementary References 37

Supplementary Methods 1

Raman Spectra of Functionalized (6,5) SWNTs

(Functionalized) single-walled carbon nanotubes (SWNTs) dispersions were drop-cast on glass substrates and Raman spectra were recorded using a Renishaw inVia Reflex confocal Raman microscope with a 532 nm laser for near-resonant excitation. More than 1000 spectra per sample were collected, averaged and baseline-corrected.

Fluorescence Lifetime Measurements (Time-Correlated Single Photon Counting, TCSPC)

Fluorescence lifetimes of luminescent defect states were determined by time-correlated single photon counting (TCSPC) as reported previously.[1] The wavelength-tunable output of a picosecond-pulsed (~6 ps pulse width) supercontinuum laser source (Fianium WhiteLase SC400) focused into a nanotube dispersion via a 50x NIR-optimized objective (N.A. 0.65, Olympus) was used to excite SWNTs at the E_{22} transition (e.g., 575 nm for (6,5) SWNTs). Emitted photons were collected with the same objective. The photoluminescence was filtered by a spectrograph (Acton SpectraPro SP2358, grating blaze 1200 nm, 150 lines mm^{-1}) and focused onto a gated InGaAs/InP avalanche photodiode (Micro Photon Devices). Arrival times of the detected photons were recorded with a time-correlated single-photon counting module (Picoharp 300, Picoquant GmbH). The instrument response function (IRF) was determined by the fast, instrument-limited photoluminescence decay at the E_{11} transition (e.g. ~1000 nm for (6,5) SWNTs). All fluorescence decay histograms were fitted with a biexponential model in a reconvolution procedure.

Normalization of Photoluminescence Spectra

To display changes in defect density (see Figure 1b-d and Figure 4a,b of the main manuscript) normalized PL spectra are presented instead of absolute emission intensities for the following experimental reasons:

(1) To stop the reaction and remove side products, the functionalization protocol includes a filtration, washing and redispersion sequence (see detailed sp^3 functionalization protocol). It is not possible to simply record the PL spectra during the reaction. As with pristine SWNTs, the yield of the redispersion step varies and depends on parameters such as sonication power,

environmental humidity and temperature. Hence, the resulting dispersions have different concentrations of the dispersed SWNTs and consequently different absolute PL intensities.

(2) While the absorption spectrum of the SWNTs is unaffected at lower levels of functionalization, samples with higher defect densities display a significant reduction of the main absorption band. Hence, the effective absorption cross-section for the functionalized SWNTs also depends on defect density preventing an accurate correction for the yield of the redispersion step.

(3) PL measurements were performed by focusing the excitation laser into a cuvette through an objective. This configuration has the advantage that the near-infrared absorption of toluene does not affect the PL spectra due to the extremely short path length within the liquid. However, even slight differences in the quality of the focus have an impact on the absolute PL intensities, while the spectrum is usually unaffected by minor changes in focus.

As a result, comparison of the absolute PL intensities across a sample series is unreliable and only normalized PL spectra are shown. However, direct and reliable values of the emission efficiencies are provided by photoluminescence quantum yield measurements (see below and Figure 2d of the main manuscript).

Photoluminescence Quantum Yield Measurements

The absolute photoluminescence quantum yield (PLQY) of pristine and functionalized nanotubes in dispersion was determined using an integrating sphere.[1,2] The SWNT dispersions were adjusted to an optical density of < 0.2 cm^{-1} at the E_{11} transition and placed in the center of an integrating sphere (LabSphere, Spectralon coating). A laser beam tuned to the E_{22} transition was directed onto the sample and the signal (scattered laser light and photoluminescence) was transmitted to the spectrometer via an optical fiber. To account for absorption of the solvent at the excitation wavelength, the same measurement was repeated with the pure solvent (toluene). PQLY was calculated as the ratio of emitted to absorbed photons. The wavelength-dependent detection efficiency and losses were corrected by recording a reference spectrum of a stabilized tungsten halogen light source with known spectral power distribution (Thorlabs SLS201/M,300-2600 nm) that was placed in front of the integration sphere.

Low and Variable Temperature Spectroscopy of SWNTs

Low-temperature photoluminescence spectra of individual, PFO-BPy-wrapped, functionalized (6,5) SWNTs embedded in polystyrene and dense films of nanotubes were recorded using a closed-cycle liquid helium optical cryostat (Montana Instruments Cryostation s50) with an adjustable temperature between 3.8 K and 300 K. The nanotube samples were excited with a continuous wave laser diode (OBIS, Coherent Inc., 640 nm) through an infrared 50x long working distance objective (Mitutoyo, N.A. = 0.42) mounted outside the cryostat. The laser power was typically ~100 µW and the polarization was adjusted with a $\lambda/2$ plate to match the orientation of individual nanotubes. PL spectra were acquired with a thermoelectrically cooled InGaAs camera (NIRvana 640ST, Princeton Instruments) mounted on a corresponding spectrograph (IsoPlane SCT-320, Princeton Instruments).

Room Temperature Spectroscopy of Individual SWNTs and Autocorrelation Measurements

Room-temperature photoluminescence and autocorrelation measurements were performed on individual functionalized (6,5) SWNTs embedded in polystyrene with a home-built confocal microscope with slip-stick positioners (ANPxy101 and ANPz102, attocube systems). A wavelength-tunable Ti:sapphire laser (Mira, Coherent) in continuous wave mode served as the excitation source and was tuned to 995 nm to be in resonance with the E_{11} transition of (6,5) SWNTs. The excitation laser was focused onto the sample with an apochromatic objective (LT-APO/IR/0.81, attocube systems) and PL was collected by the same objective and spectrally filtered using a tunable long-pass filter (TLP01-1116, Semrock). A spectrometer (Acton SP2500, Roper Scientific) coupled with a liquid-nitrogen cooled InGaAs camera (OMA V: 1024-1.7, Roper Scientific) were used to record photoluminescence spectra. For time-resolved photoluminescence and pulsed photon correlation in a standard Hanbury-Brown and Twiss setup, the sample was excited using a supercontinuum laser (SuperK EXTREME, NKT Photonics) with a 6 ps pulse width and a 78 MHz repetition rate, tuned to 995 nm by a set of spectral filters. Emission was directed onto a superconducting single photon detector (TCOPRS-CCR-SW-85, Scontel) and photon detection events were recorded using a time-correlated single-photon counting module (PicoHarp300, PicoQuant).

Detailed *sp*^{3} Functionalization Protocol

General Remarks

Dispersions of polymer-wrapped SWNTs were functionalized with *sp*^{3}-defects via a potassium *tert*-butoxide (KO^{t}Bu)-mediated coupling approach using primarily 2-haloanilines (later also indoles/thiols/phenols) in a toluene/dimethylsulfoxid (DMSO)/tetrahydrofuran (THF) mixture. While the concentrations of the reactive reagent (e.g., aniline derivative) and DMSO were kept constant, the degree of functionalization could be controlled by the amount of base (KO^{t}Bu), reaction time or temperature. SWNT dispersions (after removal of excess polymer) were used as the starting material and the nanotube concentration was always kept at 0.54 mg L^{-1} (corresponding to an E_{11} absorbance of 0.3 cm^{-1} for (6,5) SWNTs).[3]

Filtration of the nanotube dispersion and redispersion in pure solvent is used to remove excess wrapping polymer and was performed for all reactions presented in this study for higher reproducibility. However, this step is mainly needed for reactions with UV irradiation to avoid light attenuation at 365 nm due to strong absorption by the polymer. The filtration step is not necessary when the functionalization is performed in the dark and the concentration of the wrapping polymer is below ~0.3 g L^{-1}.

Reagents

All chemicals used in the described functionalization reactions were purchased from Sigma Aldrich and used without further purification:

2-iodoaniline (98%), 2-bromoaniline (97%), 2-fluoroaniline (99%), 5-fluoro-2-iodoaniline (97%), 2-iodophenol (98%), thiophenol (97%), indole (99%), potassium *tert*-butoxide (98%), dimethylsulfoxide (anhydrous, ≥99.9%), tetrahydrofuran (anhydrous ≥99.9% inhibitor free).

The quality of DMSO was found to have a significant impact on the reactivity but not on the selectivity. In order to achieve comparable reactivities as presented in this work, freshly dried DMSO is recommended. Stock solutions of KO^{t}Bu and DMSO should be stored under inert gas atmosphere, but the functionalization itself can be performed in an open flask at room temperature.

Step by Step Reaction Protocol

1. Filter polymer-wrapped SWNTs dispersion over a PTFE membrane (Merck Millipore JVWP, 0.1 µm pore size) and wash the resulting filter cake three times (each 5 minutes) with hot toluene (80 °C) to remove excess wrapping polymer.
2. Redisperse the washed filter cake by ultrasonication and adjust the SWNT concentration to an optical density of >1.0 cm^{-1} at the E_{11} absorption peak.
3. Prepare a solution of KO^tBu in dry THF under nitrogen atmosphere. The total volume of THF should result in 8.3 vol-% of the final reaction volume. The amount of KO^tBu used in this work is always given as molar equivalents (eq.) with respect to the used aryl reagent (e.g., aniline derivative). See Supplementary Table 1 for typical values.
4. Dissolve desired amount of reagent (e.g., aniline derivative) in a clear glass vial equipped with a stirring bar with appropriate amount of toluene. Note: After the addition of all components the final concentration of the reagent should be 29.30 mmol L^{-1}.
5. Add dry DMSO to the solution of the aryl compound in toluene to obtain 8.3 vol-% of DMSO in the final mixture.
6. Add the prepared KO^tBu/THF solution.
7. Add the prepared (polymer-depleted) SWNT dispersion such that the final reaction mixture has an optical density at the E_{11} transition of 0.3 cm^{-1}.
8. Mix the reaction volume thoroughly. The final ratio of toluene/DMSO/THF should be 83.3 : 8.3 : 8.3 vol-%.

It is important that the reagent (e.g., aniline derivative) and DMSO are present in the reaction mixture before the addition of KO^tBu/THF to prevent undesired side-reactions.

When functionalization is performed in the dark:

9. Protect the glass vial from light and stir the reaction mixture for the desired duration at room temperature. Reaction times can range between 15 min and 180 min.

When functionalization is performed under UV light irradiation:

10. Irradiate the glass vial with UV-light (365 nm, here SOLIS-365C, Thorlabs, 1.9 mW/mm^2) under continuous stirring. Reaction times usually vary between 10 min and 45 min.

Work up:

11. After the desired functionalization time has elapsed, pass the reaction mixture through a PTFE membrane (e.g. Merck Millipore JVWP, 0.1 mm pore size) and wash the filter cake with approximately 5 mL MeOH and 5 mL toluene on the filtration setup.
12. Redisperse the filter cake in the desired amount of toluene with a low concentration of fresh wrapping polymer (e.g. 0.1 g L^{-1}) by bath sonication for 20 min. Addition of wrapping polymer is not strictly necessary, but increases the colloidal stability of the dispersion for characterization. Note that for high defect densities (D/G^+ ratio greater than 0.2) the yield of the redispersion process starts to decline due to increasing aggregation of the functionalized SWNTs.

Example Values for Reaction Mixture

Supplementary Table 1: Exemplary values for functionalization of PFO-BPy wrapped (6,5) SWNTs with 2-iodoaniline and 2 eq. KO^{t}Bu as performed in this study.

Reagent	Amount
KO^{t}Bu (2 eq.)	78.5 mg
THF	1 mL
2-iodoaniline	76.6 mg
Toluene	7.188 mL
DMSO	1 mL
Polymer-free (6,5) SWNTs (1.28 cm^{-1} at the E_{11}) in toluene	2.812 mL
**Total reaction volume**	**12 mL**

Spectroscopic Characterization of Pristine PFO-BPy Wrapped (6,5) SWNTs

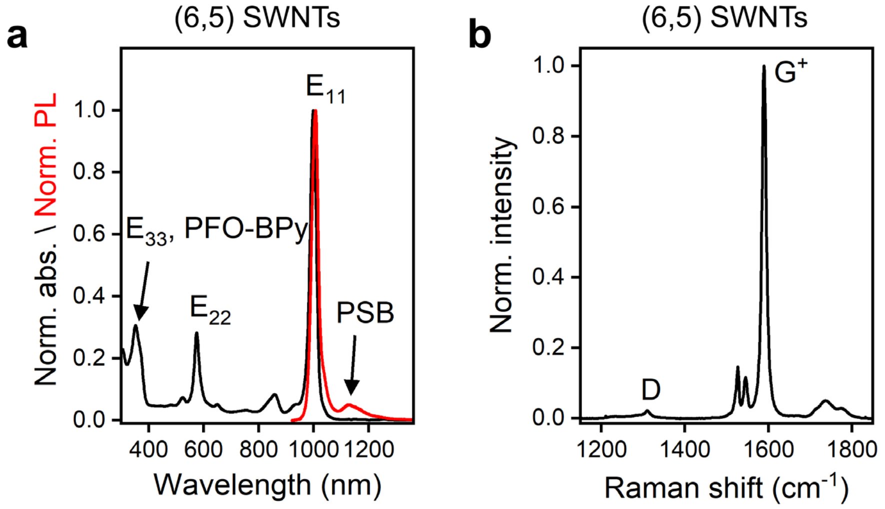

Supplementary Figure 1. a, Normalized absorption (black) and photoluminescence (red) spectra showing characteristic excitonic transitions of (6,5) SWNTs labelled as E_{11}, E_{22} and E_{33} and PSB (photoluminescence sideband). The small absorption peak below 400 nm indicates the low residual concentration of PFO-BPy after removal of excess wrapping polymer. **b**, Normalized and averaged Raman spectra (resonant excitation at 532 nm) of drop-cast pristine (6,5) SWNTs showing the G-modes (G^+, G^-) and D-mode. The G modes correspond to the longitudinal and tangential phonons of the hexagonal *sp*^{2}-carbon lattice, while the D-mode is related to disorder and defects. The ratio of their intensities (D/G^+) is used as a metric for the defect density.

Supplementary Note 1: Concentration Effects

Following the general procedure described above, (6,5) SWNTs were also functionalized using different concentrations of 2-iodoaniline and KO^{t}Bu. The ratio of 2-iodoaniline to base was kept at 1:2. As the the functionalization was mostly conducted with a large excess of reagent (58.6 – 14.65 mmoL L^{-1} 2-iodoaniline) compared to the SWNTs small changes in concentration did not significantly alter the functionalization process as shown in Supplementary Figure 2. For much lower concentrations (<10 mmoL L^{-1} 2-iodoaniline), the E_{11}*⁻ emission feature drops significantly. This is expected, because (1) the amount of reactive intermediate is greatly reduced and (2) the relative quenching of KO^{t}Bu by moisture may be increased as we perform the reaction in an open flask. At higher concentrations this quenching becomes negligible.

To further understand the effect of the concentration of 2-iodoaniline, we conducted functionalization with various concentrations of 2-iodoaniline, while the concentration of KO^{t}Bu was kept constant. This led to an increase of the ratio of 2-iodoaniline to KO^{t}Bu. Since the functionalization is still performed with large excess of the reactant compared to the SWNTs, the $E_{11}*^{-}$ emission is higher for higher 2-iodoaniline/KO^{t}Bu ratios in agreement with Figure 1c.

Concentration Effects on the Functionalization Process

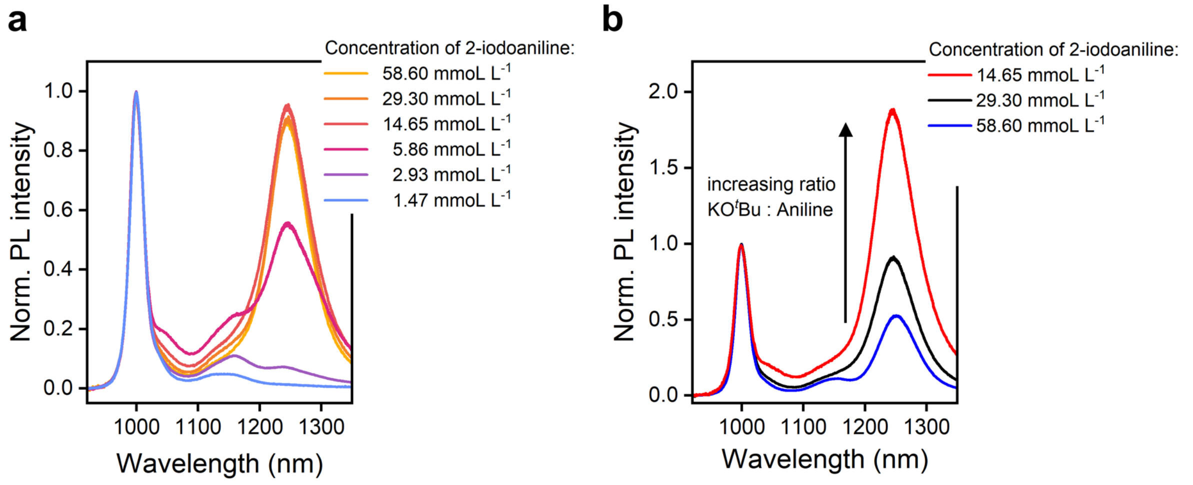

Supplementary Figure 2. a, Normalized photoluminescence spectra of (6,5) SWNTs after functionalization with different concentrations of 2-iodoaniline and 2 eq. of KO^{t}Bu for 10 minutes in the dark in toluene/DMSO/THF. **b,** Normalized photoluminescence spectra of (6,5) SWNTs after functionalization with different concentrations of 2-iodoaniline for 10 minutes in the dark in toluene/DMSO/THF. The concentration of KO^{t}Bu was kept at 58.60 mmol L^{-1}.

Functionalization with Different Aniline Derivatives

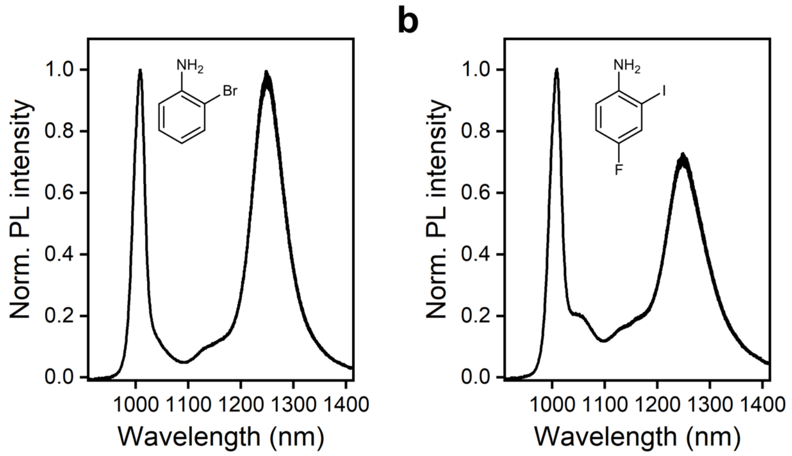

Supplementary Figure 3. a,b, Normalized photoluminescence spectra of (6,5) SWNTs after functionalization with 2-bromoaniline (**a**) and 5-fluoro-2-iodoaniline (**b**) with 2 eq. of KO*t*Bu for 30 minutes in the dark in toluene/DMSO/THF. The concentration of 2-bromoaniline and 5-fluoro-2-iodoaniline was kept constant at 29.30 mmol L^{-1}.

Raman Spectra of Functionalized (6,5) SWNTs

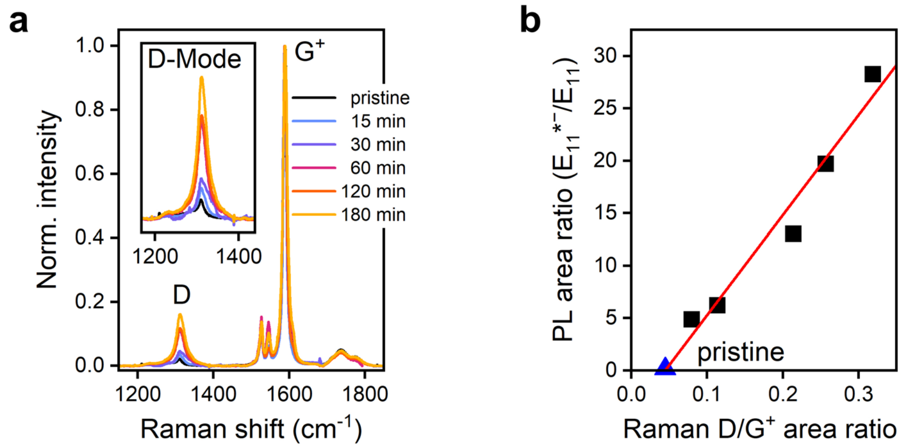

Supplementary Figure 4. a, Averaged Raman spectra of (6,5) SWNTs functionalized with 2-iodoaniline and KO*t*Bu in the dark (resulting in $E_{11}{}^{*-}$ defects) at different reaction times (corresponding to data in Figure 1d) normalized to G^+-mode. The inset shows the D-mode region. The defect-related D-mode intensity increases with reaction time. **b**, Integrated $E_{11}{}^{*-}/E_{11}$ emission ratios vs. integrated Raman D/G^+ ratios and linear fit as metric of defect density. Blue triangle: data point for pristine (6,5) SWNTs (see Supplementary Figure 1b).

Power-Dependence of $E_{11}*^{-}$ Defect State Photoluminescence

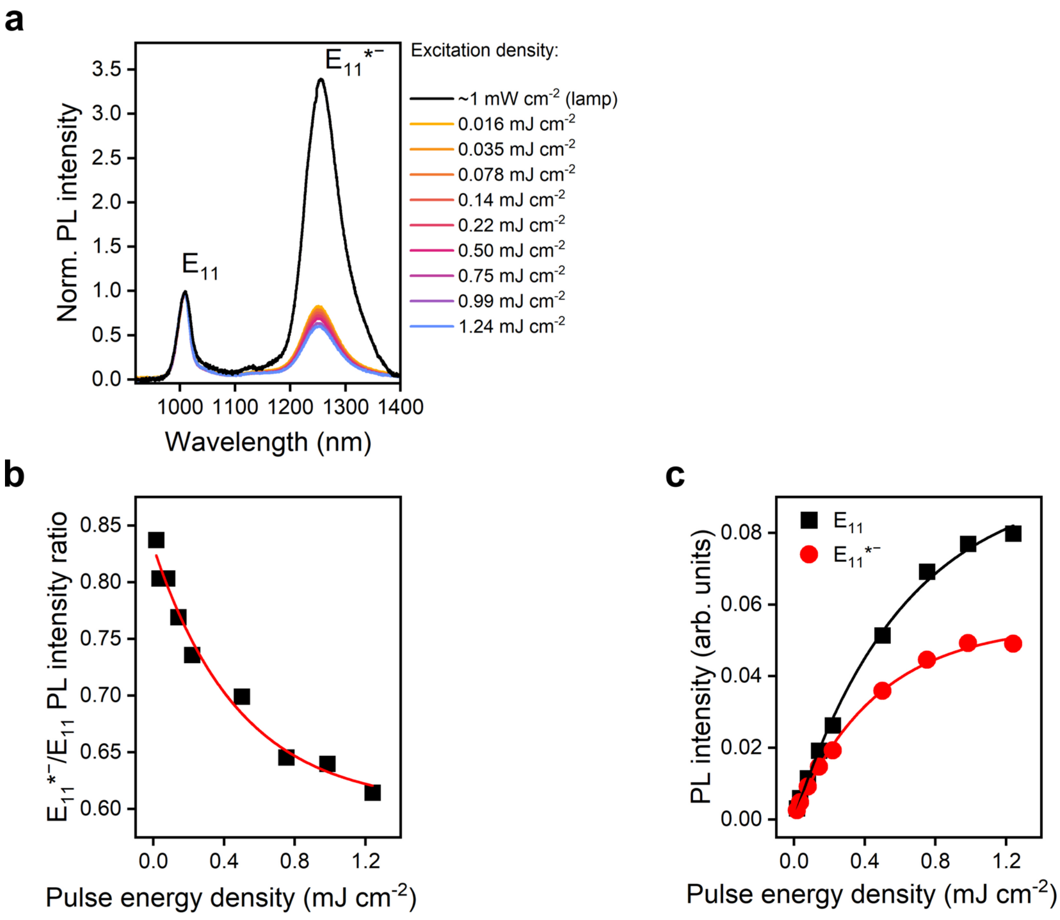

Supplementary Figure 5. a, Normalized photoluminescence spectra of (6,5) SWNTs functionalized with 2-iodoaniline recorded at different excitation (wavelength 575 nm) densities (pulsed) and under lamp illumination (black). **b**, $E_{11}*^{-}/E_{11}$ intensity ratios vs. pulse energy density. **c**, Absolute $E_{11}*^{-}$ (red) and E_{11} (black) intensity vs. pulse energy density (lines are guides to the eye).

Defect State Power-Dependence Depending on Optical Trap Depth

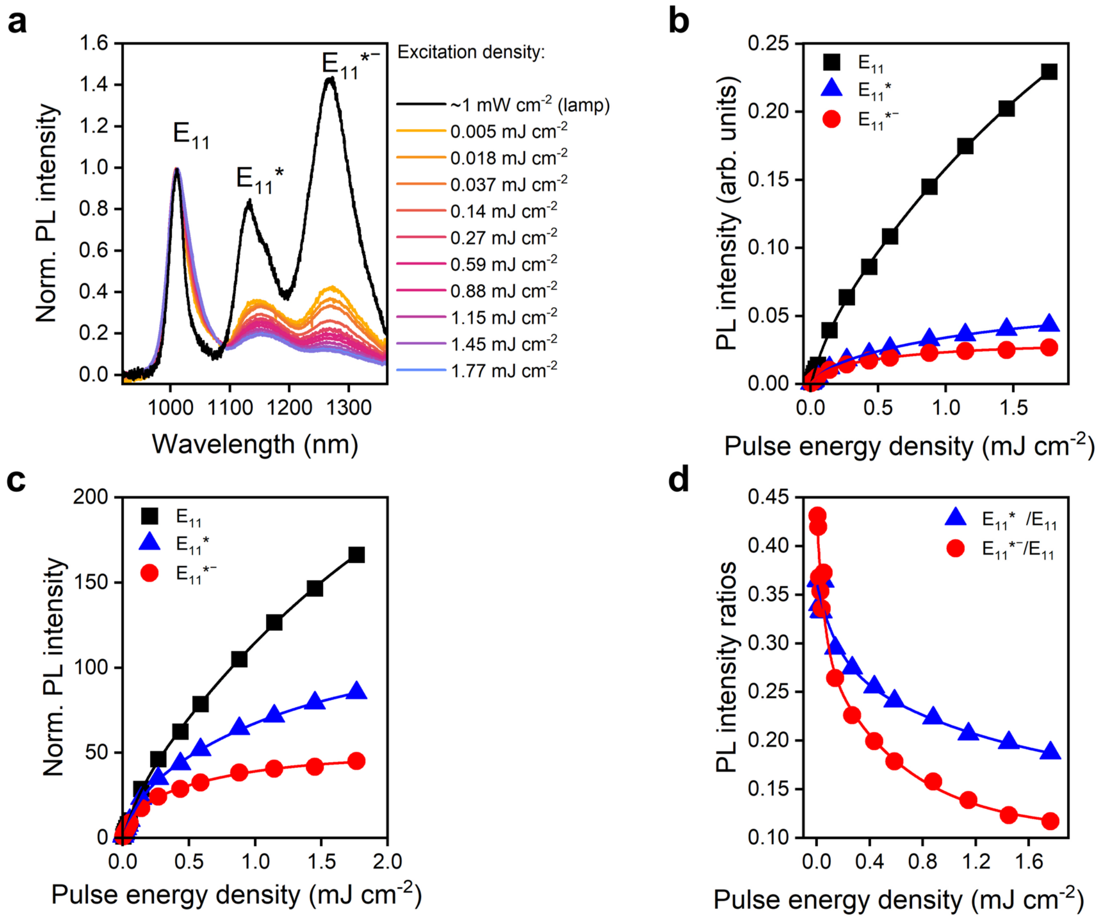

Supplementary Figure 6. a, Normalized PL spectra of (6,5) SWNTs functionalized with 2-iodoaniline under UV light irradiation yielding two defect emission bands with different optical trap depths labelled as $E_{11}*$ and $E_{11}*^{-}$. Spectra were recorded at different excitation (wavelength 575 nm) densities (pulsed) and under lamp illumination. **b**, Absolute $E_{11}*^{-}$ (red), $E_{11}*$ (blue) and E_{11} (black) intensity vs. pulse energy density. **c**, Intensity of $E_{11}*^{-}$ (red), $E_{11}*$ (blue) and E_{11} (black) emission vs. pulse energy density normalized to intensity at lowest laser power. **d**, $E_{11}*^{-}/E_{11}$ (red) and $E_{11}*/E_{11}$ (blue) intensity ratios vs. pulse energy density.

Defect State Photoluminescence Decay Time Traces

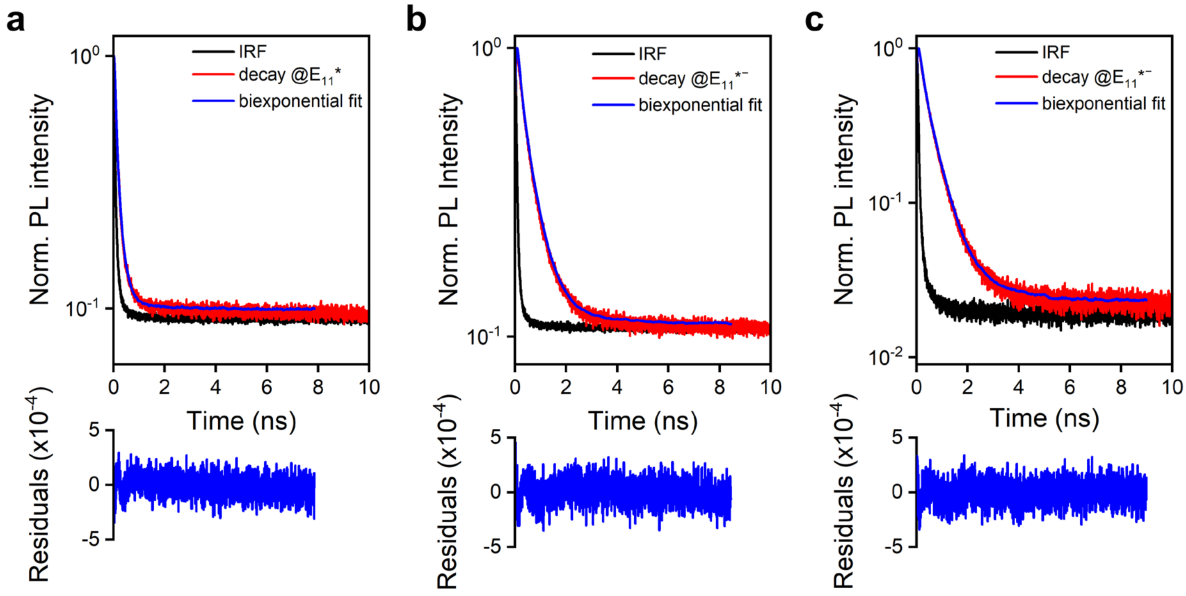

Supplementary Figure 7. a-b, TCSPC histograms of the photoluminescence decay (red) of (6,5) SWNT functionalized with 2-iodoaniline under UV light irradiation measured at the E_{11}^{*} (**a**) and E_{11}^{*-} (**b**) emission maximum. **c**, TCSPC histogram of the photoluminescence decay (red) of (6,5) SWNT functionalized with 2-iodoaniline in the dark (only E_{11}^{*-}). Note that photoluminescence decays of **a** and **b** were measured on the same sample. In all cases the time traces were fitted as a biexponential decay (blue) with a reconvolution method using the fast E_{11} decay as the instrument response function (IRF, black). Residuals of the fits are shown below.

Overview of Different Defect State Photoluminescence Lifetimes

Supplementary Table 2. Extracted short and long lifetime components (τ_{short}, τ_{long}) and corresponding normalized amplitudes (A_{short}, A_{long}) for defect emission from (6,5) SWNTs functionalized with various reagents depending on the optical trap depth (energy offset between E_{11} and defect emission). Corresponding defect emission bands are indicated as E_{11}^{*} and E_{11}^{*-}.

Reagent	Optical trap depth (meV)	τ_{short} (ps)	τ_{long} (ps)	A_{short} (%)	A_{long} (%)
2-iodoaniline	247 (E_{11}^{*-})	134	568	54.0	46.0
2-iodoaniline	136 (E_{11}^{*})	33	179	86.7	13.3
2-bromoaniline	241 (E_{11}^{*-})	127	589	45.5	54.5
5-fluoro-2-iodoaniline	244 (E_{11}^{*-})	108	555	55.4	44.6

Supplementary Note 2: Temperature Dependence of Defect State Photoluminescence

To investigate the impact of temperature on defect emission we recorded PL spectra of thin films of (6,5) SWNTs functionalized with 2-iodoaniline (in the dark and under UV illumination) from 4 K to 330 K (Supplementary Figures 8 and 9) in an optical cryostat as well as of dispersions between 278 K and 308 K (Supplementary Figures 10 and 11) in a Peltier-based temperature-controlled cuvette holder (Fluorolog). Note that the relative intensity of defect emission varies strongly between PL measurements of dispersions and thin films (see Supplementary Figures 9 and 11). This effect can be assigned to the power-dependence of the defect state emission compared to E_{11}, which is more pronounced for $E_{11}{}^{*-}$ defects (see Supplementary Figure 6).

Starting at 4 K, the nanotube thin films showed a relative increase in defect emission with increasing temperature (both for $E_{11}{}^{*}$ and $E_{11}{}^{*-}$) reaching a maximum between 180 K and 220 K. This increase in defect emission correlates well with the model of a potential barrier around the defect site as proposed by Kim et al.[4] In this context, the increase in defect emission is attributed to a higher exciton trapping efficiency. After reaching this maximum, the defect emission steadily decreased again. This decrease in defect emission was investigated in more detail for dispersions and closer to room temperature.

The temperature-dependent distribution of localized and mobile excitons is commonly associated with a certain detrapping energy of the localized excitons, which is however quite different from the optical trap depth. As described by Kim et al. the thermal detrapping energy $\Delta E_{\mathrm{thermal}}$ can be determined from a van't Hoff plot of the ratio of the integrated PL intensities ($I_{E_{11}}$ and $I_{E_{11}^{*}}$ or $I_{E_{11}}$ and $I_{E_{11}^{*-}}$) at different temperatures (T):[5]

$$\frac{I_{E_{11}}}{I_{E_{11}^{*}}} \propto e^{-\left(\frac{\Delta E_{thermal}}{kT}\right)} \tag{1}$$

$$\ln\left(\frac{I_{E_{11}}}{I_{E_{11}^{*}}}\right) = -\frac{\Delta E_{thermal}}{kT} + A \tag{2}$$

where k is the Boltzmann constant and A is a correction factor. The van't Hoff plots for the $E_{11}{}^{*}$ and $E_{11}{}^{*-}$ emission bands display good linear fits to the data and extraction of $\Delta E_{\mathrm{thermal}}$. According to this analysis the $E_{11}{}^{*}$ defect exhibits a detrapping energy of approximately

79 meV, while the apparent detrapping energy for $E_{11}*^-$ is surprisingly low with ~25 meV (see Supplementary Figures 10 and 11). Note that for the determination of the detrapping energy of $E_{11}*^-$ defects, samples with different defect densities were used. Kim *et al.* previously reported that the detrapping energy increases at higher defect density.[5] While a similar trend can be observed here, the detrapping energy of $E_{11}*^-$ defects increases only slightly from 23 to 27 meV. Such a low detrapping energy is in clear contrast to the increased defect state PL lifetime of $E_{11}*^-$. Previously, the strong correlation between defect state lifetime and optical trap depth was suggested to originate from phonon-assisted thermal detrapping.[6] Thus, we expect that a different temperature-dependent non-radiative decay mechanism, such as multiphonon decay, is dominant for $E_{11}*^-$ defects within the high temperature range. This finding further highlights the complex relationship between optical trap depths and thermal detrapping.

Temperature Dependent Defect State PL in Thin Films

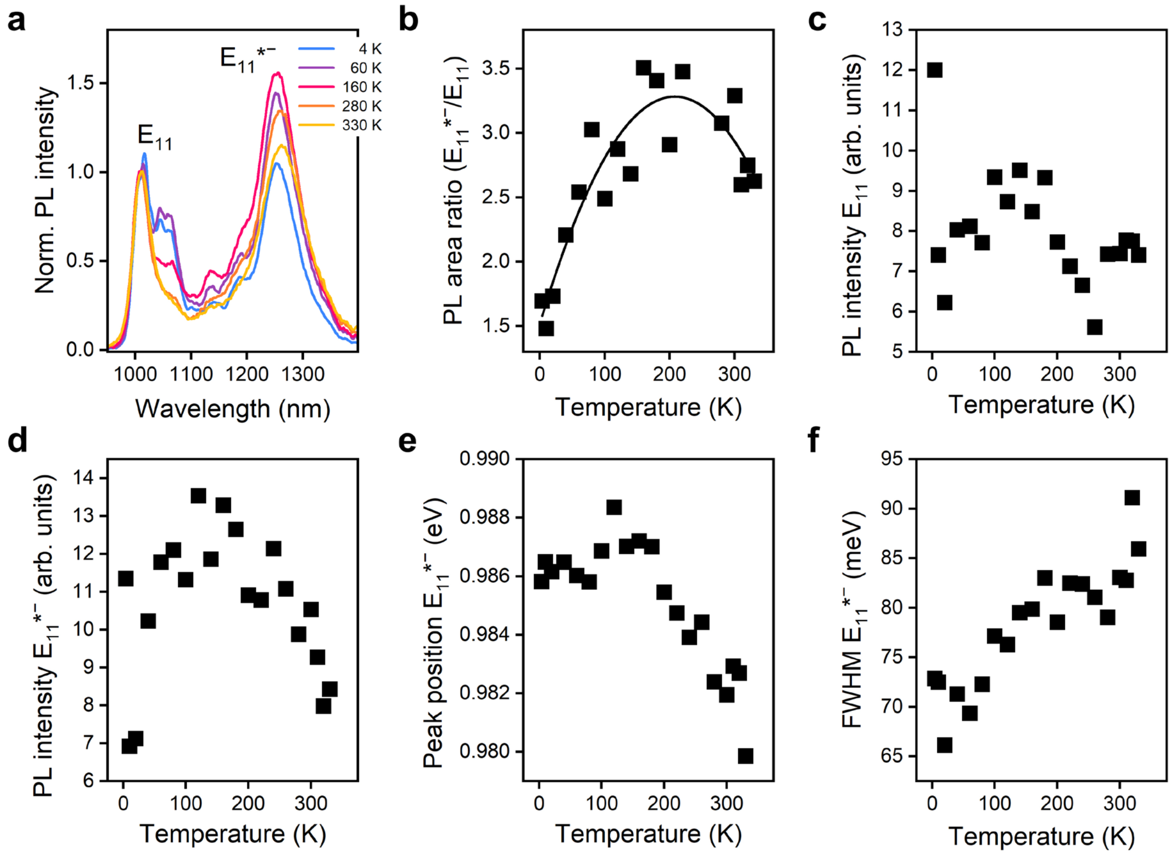

Supplementary Figure 8. a, Temperature-dependent (4 K – 330 K) defect state photoluminescence of thin films of (6,5) SWNTs functionalized with 2-iodoaniline in the dark. **b**, Integrated $E_{11}*^{-}/E_{11}$ emission ratios vs. temperature. The defect emission ($E_{11}*^{-}$) reaches a maximum around 200 K relative to the E_{11} emission. Solid line are guides to the eye. **c-f**, Temperature-dependent change of absolute E_{11} emission (**c**), absolute $E_{11}*^{-}$ emission (**d**), peak position of $E_{11}*^{-}$ emission (**e**) and full width at half maximum (FWHM) of $E_{11}*^{-}$ emission peaks (**f**).

Note, emission features around 1050 nm are not exclusively observed for functionalized SWNT but also for pristine SWNTs as reported by Kadria-Vili *et al.* and commonly labelled as Y_1 band.[7] It is assumed that this feature originates from defects that are unintentionally introduced during processing of SWNTs. The observed intensity can therefore vary even for ("pristine") SWNTs before functionalization. The functionalization of SWNTs represents an additional processing step with varying reaction times, sonication and heating steps (for SWNT films as presented in Supplementary Figure 8a) and thus can lead to even stronger batch-to-batch variations. The temperature dependence of these features is not yet understood.

Effect of Optical Trap Depth on Temperature Dependent Defect PL (Film)

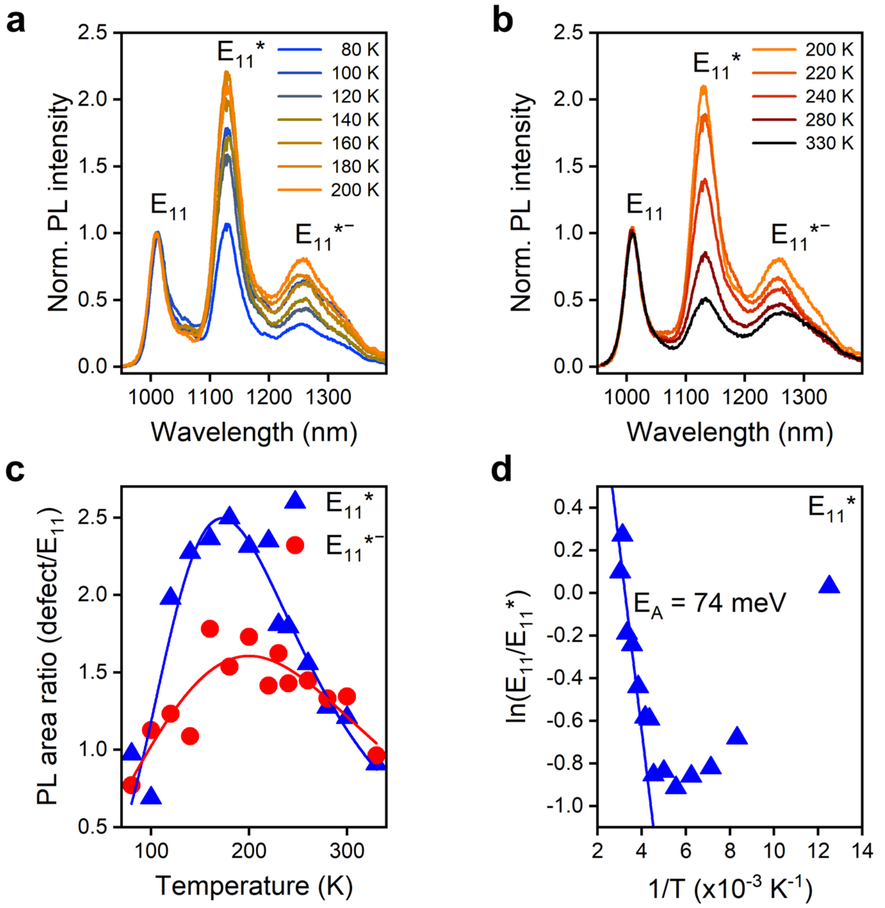

Supplementary Figure 9. a-b, Temperature dependent defect state PL of films of (6,5) SWNTs functionalized with 2-iodoaniline under UV light irradiation. Defect state PL is increasing relative to E_{11} emission up to 200 K (**a**) and decreasing from 200 K to 330 K (**b**) **c**, Integrated E_{11}*/E_{11} (blue) and E_{11}^{*-}/E_{11} (red) emission ratios vs. temperature. Solid lines are guides to the eye. **d**, van't Hoff plot for the E_{11}* emission band and linear fit to the data. A detrapping energy ($\Delta E_{thermal}$) of 74 meV was determined.

Temperature Dependent Defect State PL in SWNT Dispersion

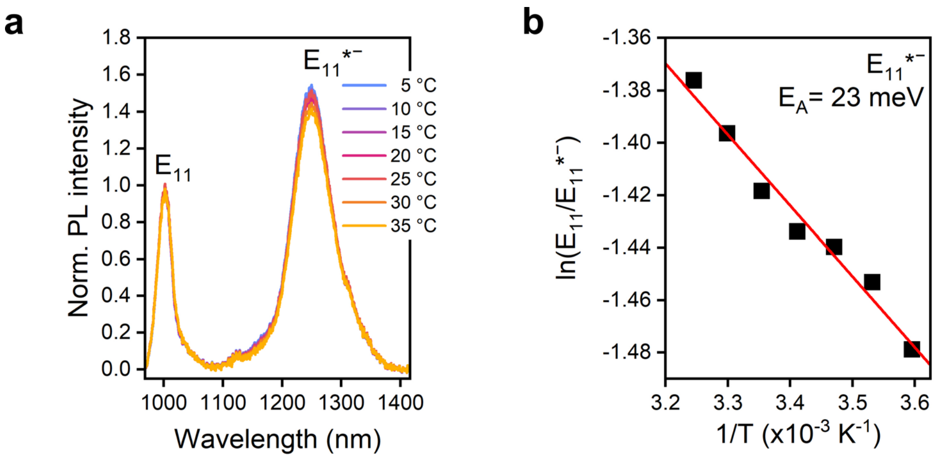

Supplementary Figure 10. a, Temperature-dependent PL spectra of dispersions (excitation with Xenon lamp) of (6,5) SWNTs functionalized with 2-iodoaniline in the dark. **b**, van't Hoff plot for the $E_{11}*^{-}$ emission band and linear fit to the data. A detrapping energy ($\Delta E_{thermal}$) of 23 meV was extracted. Note that the defect concentration of the functionalized (6,5) SWNTs in this measurement was lower compared to Supplementary Figure 8 (thin film) to ensure dispersion stability.

Effect of Optical Trap Depth on Temperature Dependent Defect PL (Dispersion)

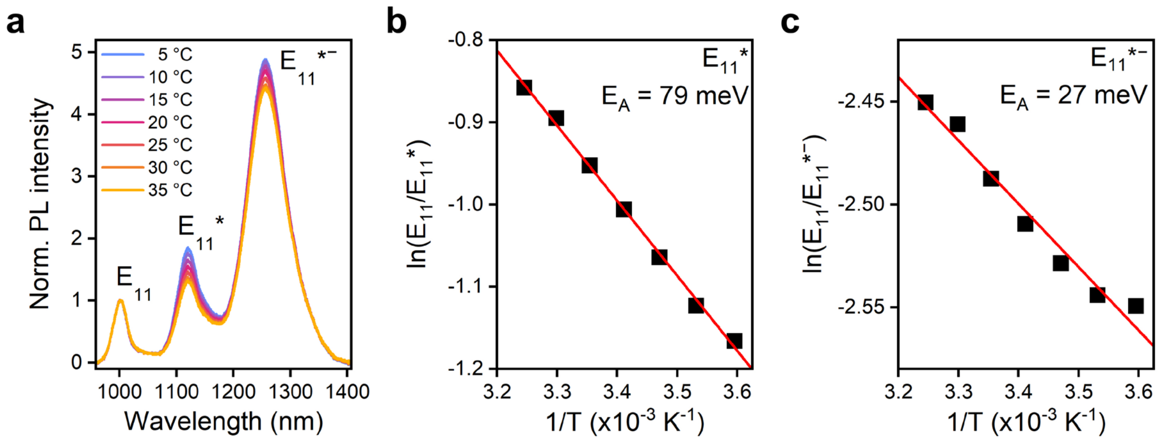

Supplementary Figure 11. **a**, Temperature-dependent photoluminescence spectra (excitation with Xenon lamp) of a dispersion of (6,5) SWNTs functionalized with 2-iodoaniline by UV light irradiation. **b**, van't Hoff plot for the $E_{11}*$ emission band and linear fit to the data. **c**, van't Hoff plot for the $E_{11}*^{-}$ emission band and linear fit to the data. Detrapping energies ($\Delta E_{thermal}$) of 79 meV (**b**) and 27 meV (**c**) were extracted. Note that the same functionalized SWNTs were used to prepare the film in Supplementary Figure 9.

Cumulative Photoluminescence Spectrum from Individual (6,5) SWNTs

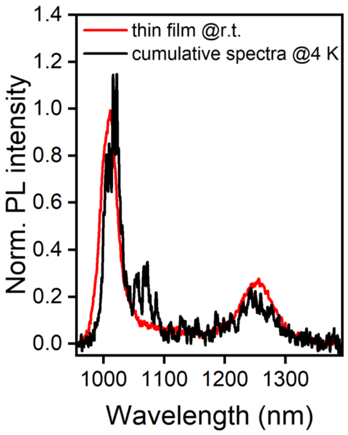

Supplementary Figure 12. Cumulative photoluminescence spectrum of 62 measured individual nanotubes (spots) at 4 K (black) representing the average signal of a large number of functionalized (6,5) SWNTs. The cumulative spectrum (black line) fits the spectrum measured for a thin film of the same functionalized (6,5) SWNTs at room temperature (r.t., red line). The higher signal at 1060 nm at low temperature is assumed to originate from defects unintentionally introduced during initial processing of SWNTs (dispersion etc.). Their number and emission intensity vary even for untreated ("pristine") SWNTs.[7]

Absorption Characteristics of Polymer Wrapped Large Diameter SWNTs

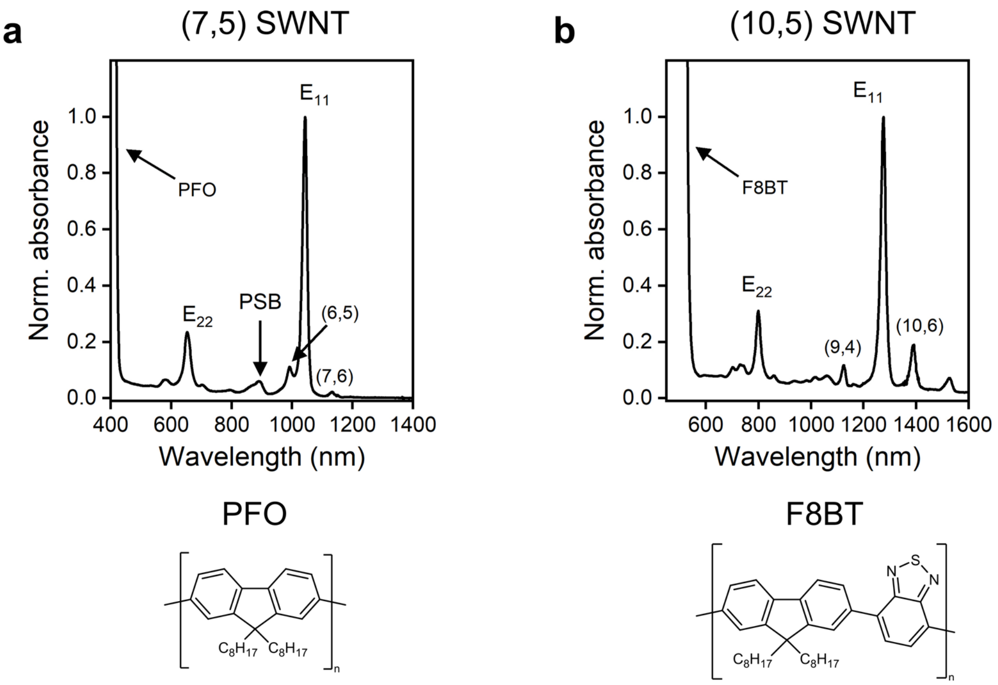

Supplementary Figure 13. a, Normalized absorption spectra of (7,5) SWNTs selectively dispersed in toluene with PFO. **b**, Normalized absorption spectra of (10,5) SWNTs selectively dispersed in toluene with F8BT. Characteristic nanotube transitions are indicated as E_{11}, E_{22} and PSB (phonon sideband). Additional SWNT species and absorption bands of the wrapping polymers are indicated.

Characterization of (7,5) SWNTs Functionalized with 2-Iodoaniline

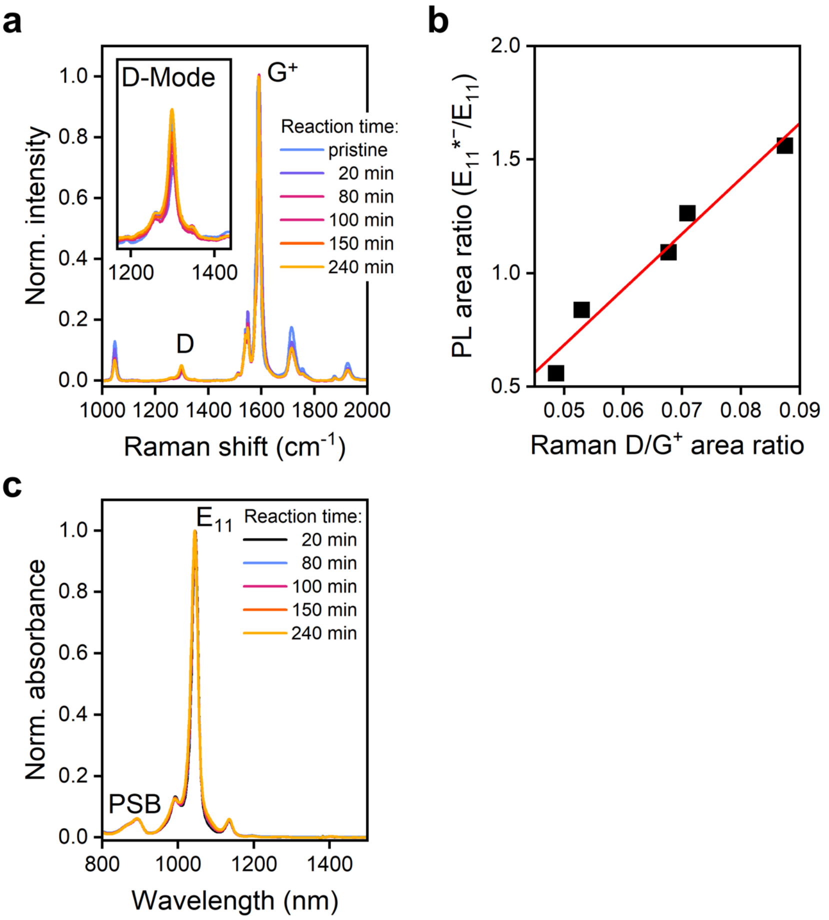

Supplementary Figure 14. a, Averaged Raman spectra of (7,5) SWNTs functionalized with 2-iodoaniline and after different reaction times. Inset: zoom-in on the D-mode region. **b**, Integrated $E_{11}*^{-}/E_{11}$ emission area ratios vs. integrated Raman D/G^{+} ratios as a metric for defect density. **c**, Absorption spectra of (7,5) SWNTs functionalized with 2-iodoaniline after different reaction times. The defect density is still too low to observe significant defect absorption.

Diameter-Dependent Defect Properties of Functionalized SWNTs

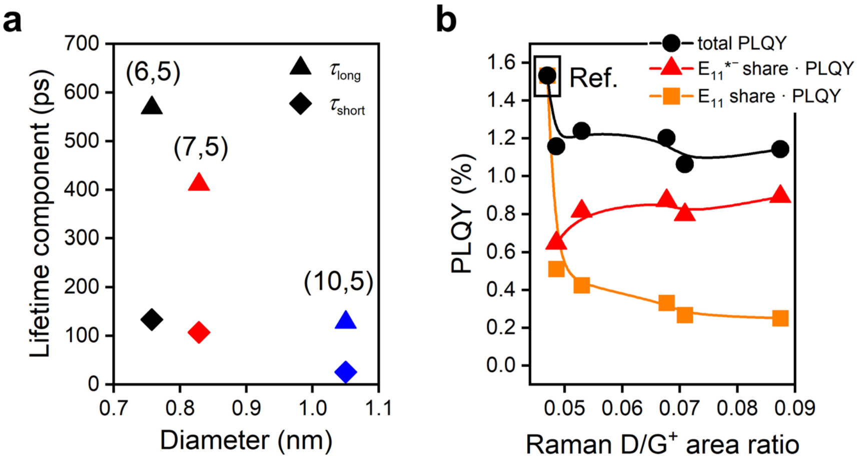

Supplementary Figure 15. **a**, Photoluminescence lifetimes (short and long component) of $E_{11}*^{-}$ defects of polymer-wrapped (6,5), (7,5) and (10,5) SWNTs decreasing with increasing SWNT diameter. **b**, Photoluminescence quantum yield (PLQY) of (7,5) SWNTs functionalized with 2-iodoaniline (total and spectral contributions) vs. integrated Raman D/G^{+} ratios as metric for defect density.

Supplementary Note 3: Mechanistic Considerations

Reference and control experiments revealed that defect emission bands located at 1130-1180 nm (E_{11}*) and ~1250 nm ($E_{11}*^{-}$) appear for various reaction conditions (see Supplementary Tables 3 and 4). As the functionalization process involves multiple components, solvents and reagents, the role and impact of each shall be summarized and discussed briefly here. It should also be noted that preference for specific defect configurations by introducing sp^3-defects in close proximity to already existing defect site may also play a minor role.[8]

Toluene:

Toluene was chosen as the main solvent because polymer-sorting of SWNTs is typically performed in this medium and the employed aniline derivatives show good solubility. This solvent choice facilitates the direct functionalization after the SWNT sorting process without further processing steps and ensures stable dispersions.

THF:

THF can be used to drastically speed up the rate of the functionalization reaction. While polymer-wrapped SWNTs remain stable in this solvent, the solubility of KO^{t}Bu is greatly increased. An enhanced reactivity can be observed by comparing the reaction times of functionalization performed in toluene *vs*. those in THF. Hence, small fractions (8.3 vol%) of THF were added to the toluene reaction mixture in the standard protocol.

Potassium *tert*-butoxide (KO^{t}Bu):

While KO^{t}Bu represents a key reagent in many organic transition-metal-free coupling reactions[9-11] it has been applied for carbon nanotube chemistry only once to the best of our knowledge.[12] Upon combining aniline derivatives with the base KO^{t}Bu, fast deprotonation of the amine group (or other acidic protons) is expected. The resulting anion may then proceed to react with the nanotube. Increasing the amount of base shifts the equilibrium of this deprotonation step and consequently increases the rate of functionalization.

It is important to note that while KO^tBu is most commonly used as a strong organic base, it can also form radicals in the presence of appropriate electron acceptors.[11] As shown by the control reactions performed in this study, KO^tBu itself is able to lead to emissive defect states in the $E_{11}*$ and $E_{11}*^-$ region without the presence of an aniline derivative. Thus, while the exact interactions with carbon nanotubes are unknown, it has the potential to follow nucleophilic as well as radical reaction paths with the nanotubes, possibly leading to $E_{11}*$ and $E_{11}*^-$ emission bands.

<u>Dimethylsulfoxide (DMSO) / Dimsyl anion:</u>

DMSO is a common co-solvent for KO^tBu and is known to dramatically increase its basicity.[13] Hence, when functionalization is performed in the dark, the addition of DMSO may shift the equilibrium of deprotonated aniline derivatives resulting in increased functinalization rates and higher selectivity towards $E_{11}*^-$ defects. Furthermore, in the presence of small amounts of a base, a dimsyl anion can be formed that behaves like a typical carbanion and represents a strong organic base.[14] Thus, in the absence of aniline derivatives it can potentially initiate nucleophilic reaction paths leading to $E_{11}*^-$ defects with high selectivity.

Upon UV light excitation (~350 nm) the dimsyl anion can act as an electron donor and radical source.[15] Under UV illumination in the presence of DMSO we observed $E_{11}*$ emission bands, which we associate with such a radical reaction path.

Additionally, weak $E_{11}*$ bands were observed when the functionalization was performed without the addition of KO^tBu under UV illumination. The absence of $E_{11}*^-$ defects is consistent with the proposed functionalization mechanism as no nucleophilic species can be formed.

<u>Aniline Derivatives:</u>

The addition of 2-iodoaniline or 2-fluoroaniline was found to greatly increase the functionalization rate for $E_{11}*$ and $E_{11}*^-$ defects when THF was used as a solvent. When functionalization is performed in toluene, vastly different reactivities and selectivities towards $E_{11}*$ and $E_{11}*^-$ defects were found compared to functionalization in the absence of aniline derivatives. Thus, a functionalization path via aniline intermediates is highly likely. It has to

be noted that E_{11}* defects were observed for functionalization with 2-fluoroaniline, thus a dehalogenation step is not necessary for the introduction of E_{11}* defects.

UV-Light Illumination:

UV-light illumination was found to increase the reaction rate for the introduction of E_{11}* as well as E_{11}*$^{-}$ defects. The latter is expected to originate partially from heating effects during the illumination process. Importantly, no reaction of the wrapping polymer with SWNTs was observed upon illumination.

General Remarks about the Importance of the Base

Several control and test reactions highlight the importance of the base in this reaction system. While a base that purely engages in a nucleophilic reaction pathway is desirable, the following aspects should be considered before testing alternative bases:

1. The highest selectivity towards the introduction of sp^3-defects showing E_{11}*$^{-}$ emission was observed for anilines, which are very poor acids with a pk_a of 28.7 (2-fluoroaniline in DMSO[16]), thus a strong base is needed for effective formation of the reactive aniline anion.
2. Strong bases such as alkyl lithium compounds lead to significant side-wall functionalization of SWNTs[17-19] and may not follow the desired functionalization path.
3. The employed base should not act as reducing agent as this can lead to a Billups-Birch type functionalization.[20]
4. To ensure high reproducibility it would be beneficial to perform the functionalization process under ambient conditions. Thus, bases that are highly sensitive toward oxygen and water should be avoided.

In summary, KO^{t}Bu in DMSO as co-solvent represents an excellent system as it is a strong base (pk_a 32.2), a poor reducing agent and bench-stable in the dark.

XPS of (6,5) SWNTs Functionalized with 5-Fluoro-2-iodoaniline

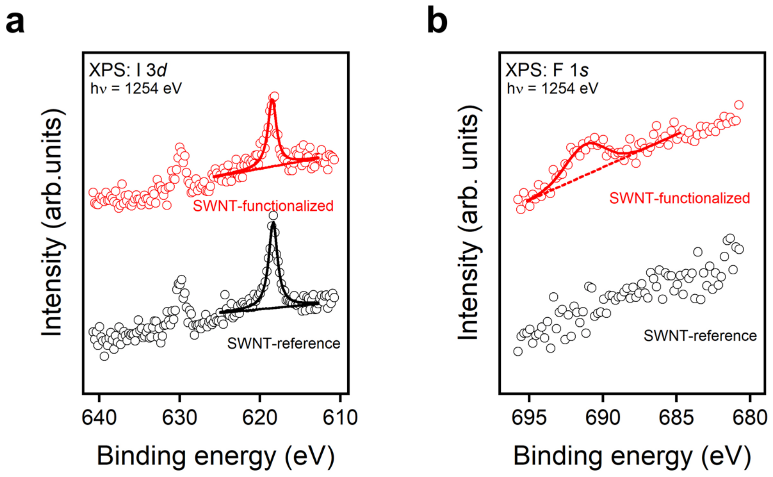

Supplementary Figure 16. a-b, I 3*d* **(a)** and F 1*s* **(b)** X-ray photoemission spectra of thin films of (6,5) SWNTs after reaction with 5-fluoro-2-iodoaniline with (red, high degree of functionalization) and without (black; reference) addition of KO*ᵗ*Bu as initiator. Solid lines represent peak fits. The observed binding energies of F 1*s* and I 3*d* can shift slightly due to charging effects, which cannot be excluded completely for thin films even on conducting substrates. No change in PL was observed for the reference compared to a pristine sample, indicating lack of functionalization. Note that 5-fluoro-2-iodoaniline could potentially undergo polymerization reactions in the presence of KO*ᵗ*Bu. The iodine signal for the reference may result from physisorbed iodine.

Reference Experiments with (6,5) SWNTs in Toluene

Supplementary Table 3. Control reactions were performed under various conditions with (6,5) SWNTs in toluene without (red cross) and with aryl reactant (2-iodoaniline or 2-fluoroaniline), with or without UV irradiation, different KO^{t}Bu concentrations, and with or without addition of THF and DMSO to increase KO^{t}Bu solubility. The resulting photoluminescence spectra are shown and observations are summarized.

Aryl reactant	Reaction conditions	PL Spectrum	Comments
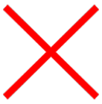	30 min<br>irradiation at 365 nm	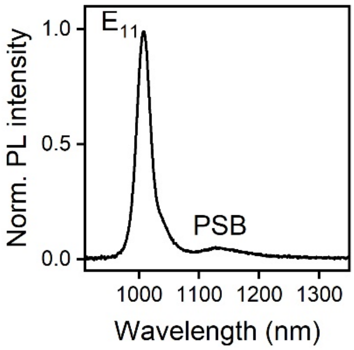	no functionalization
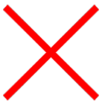	10 min<br>irradiation at 365 nm<br>58.60 mmol L^{-1} KO^{t}Bu<br>8.3 vol% THF	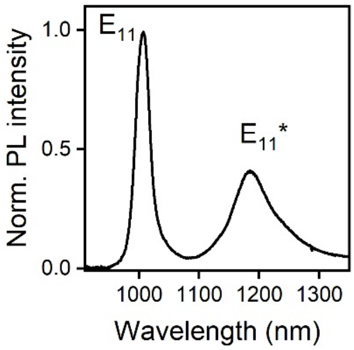	low degree of functionalization,<br>defect emission feature at 1185 nm
2-iodoaniline<br>29.30 mmol L^{-1} 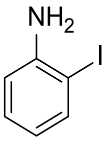	10 min<br>irradiation at 365 nm<br>58.60 mmol L^{-1} KO^{t}Bu<br>8.3 vol% THF	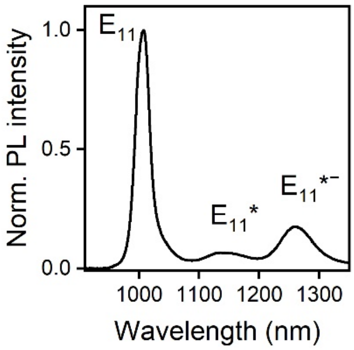	low degree of functionalization,<br>higher selectivity towards E_{11}^{*-} emission compared to reaction without 2-iodoaniline (see above)

<table>
<tr><th>Aryl reactant</th><th>Reaction conditions</th><th>Spectrum</th><th>Comments</th></tr>
<tr><td>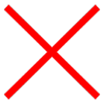</td><td>30 min
dark
58.60 mmol L^{-1} KO^tBu
8.3 vol% THF</td><td>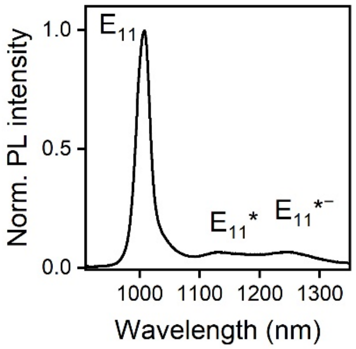</td><td>low degree of functionalization</td></tr>
<tr><td>2-iodoaniline
29.30 mmol L^{-1}
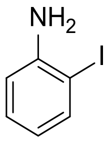</td><td>30 min
irradiation at 365 nm
14.65 mmol L^{-1} KO^tBu
8.3 vol% THF</td><td>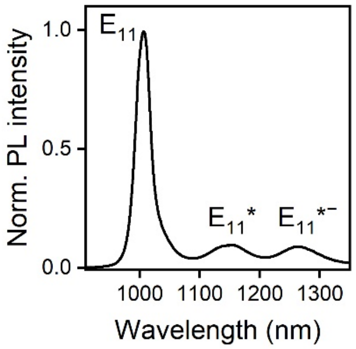</td><td>low degree of functionalization,
E_{11}* can be identified as additional defect emission band</td></tr>
<tr><td>2-iodoaniline
29.30 mmol L^{-1}
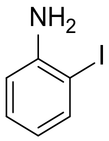</td><td>30 min
dark
58.60 mmol L^{-1} KO^tBu
8.3 vol% THF</td><td>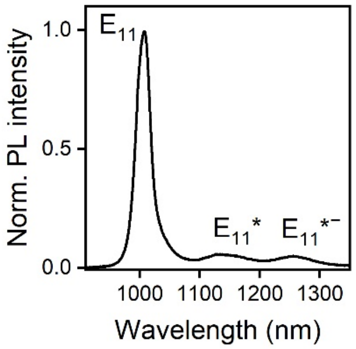</td><td>low degree of functionalization</td></tr>
</table>

Aryl reactant	Reaction conditions	Spectrum	Comments
2-iodoaniline<br>29.30 mmol L-1<br>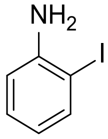	30 min<br>dark<br>58.60 mmol L-1 KOtBu<br>8.3 vol% THF<br>8.3 vol% DMSO	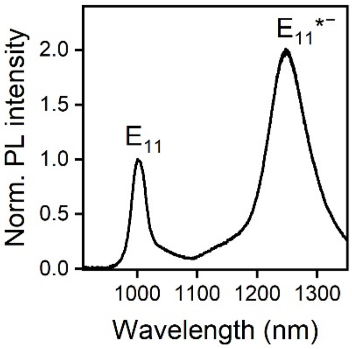	high degree of functionalization,<br>addition of DMSO leads to a dramatic increase in reactivity,<br>high selectivity towards E11*− emission
2-iodoaniline<br>29.30 mmol L-1<br>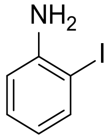	15 min<br>irradiation at 365 nm<br>21.98 mmol L-1 KOtBu<br>8.3 vol% THF<br>8.3 vol% DMSO	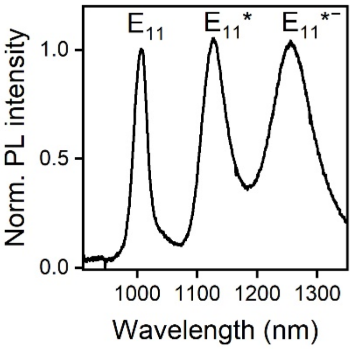	high degree of functionalization,<br>addition of DMSO leads to a dramatic increase in reactivity,<br>both E11* and E11*− emission band are prominent
2-fluoroaniline<br>29.30 mmolL-1<br>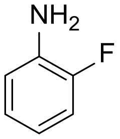	30 min<br>irradiation at 365 nm<br>21.98 mmol L-1 KOtBu<br>8.3 vol% THF<br>8.3 vol% DMSO	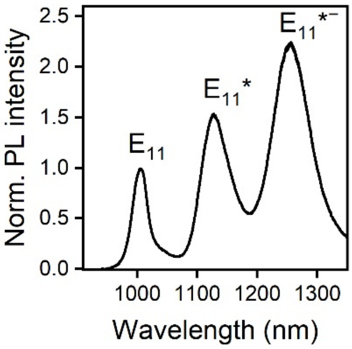	high degree of functionalization,<br>introduction of E11* defect emission can also occur without dehalogenation

Reference Experiments with (6,5) SWNTs in THF

Supplementary Table 4. Control reactions were performed under various conditions with (6,5) SWNTs in THF without (red cross) and with aryl reactant (2-iodoaniline or 2-fluoroaniline), with or without UV irradiation, different KO^{t}Bu concentrations, and with or without addition of DMSO. The resulting photoluminescence spectra are shown and observations are summarized.

Aryl reactant	Reaction conditions	Spectrum	Comments
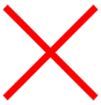	10 min<br>irradiation at 365 nm	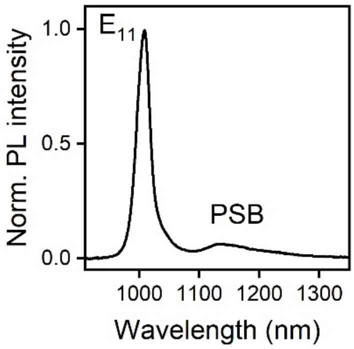	no functionalization
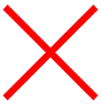	10 min<br>irradiation at 365 nm<br>8.3 vol% DMSO	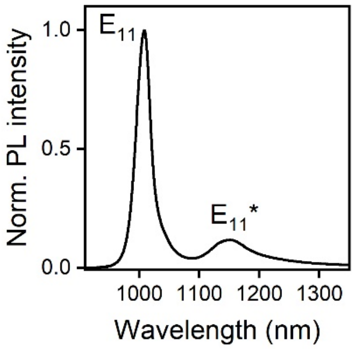	Very low degree of functionalization,<br>defect emission feature at 1151 nm
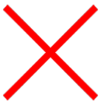	5 min<br>dark<br>58.60 mmol L^{-1} KO^{t}Bu<br>8.3 vol% DMSO	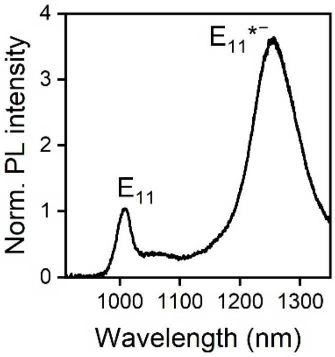	High degree of functionalization despite absence of aniline derivative,<br>higher reactivity compared to functionalization in toluene

<table>
<tr><th>Aryl reactant</th><th>Reaction conditions</th><th>Spectrum</th><th>Comments</th></tr>
<tr><td>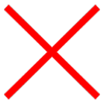</td><td>10 min
irradiation at 365 nm
58.60 mmol L^{-1} KO^{t}Bu</td><td>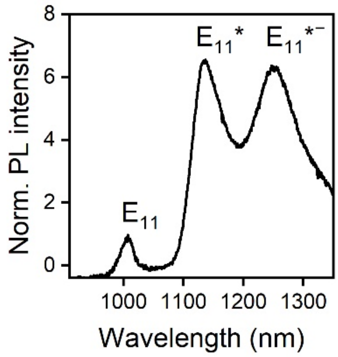
</td><td>very high degree of functionalization,
higher reactivity compared to functionalization in toluene</td></tr>
<tr><td>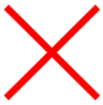</td><td>10 min
irradiation at 365 nm
14.65 mmol L^{-1} KO^{t}Bu</td><td>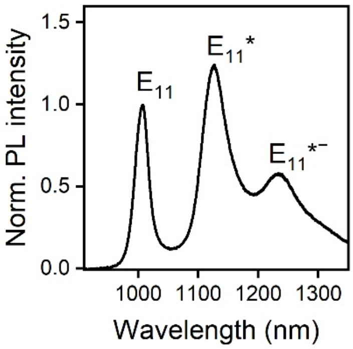
</td><td>medium degree of functionalization,
decreasing reactivity with decreasing amount of KO^{t}Bu (see above),
large E_{11}* contribution</td></tr>
<tr><td>2-iodoaniline
29.30 mmol L^{-1}
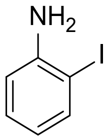
</td><td>10 min
irradiation at 365 nm
14.65 mmolL^{-1} KO^{t}Bu</td><td>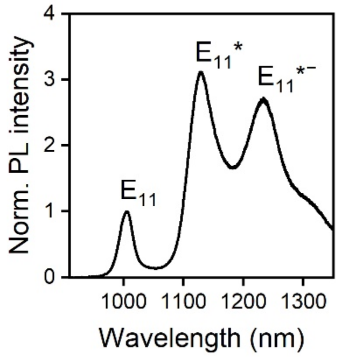
</td><td>very high degree of functionalization,
higher reactivity compared to functionalization without 2-iodoaniline (see above)</td></tr>
</table>

Aryl reactant	Reaction conditions	Spectrum	Comments

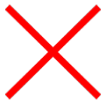

10 min

dark

58.60 mmol L^{-1} KO^{t}Bu

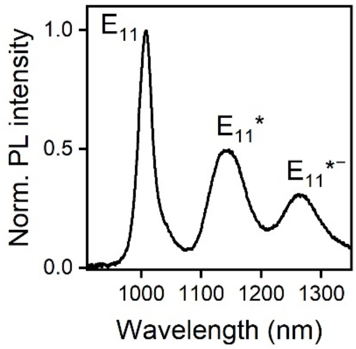

low degree of functionalization,

introduction of E_{11}* defect emission bands is not only driven by UV-light

2-iodoaniline

29.30 mmol L^{-1}

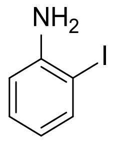

10 min

dark

58.60 mmol L^{-1} KO^{t}Bu

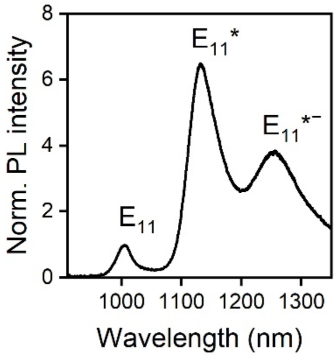

very high degree of functionalization,

higher reactivity compared to functionalization without 2-iodoaniline (see above)

2-fluoroaniline

29.30 mmol L^{-1}

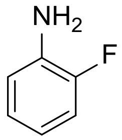

10 min

dark

58.60 mmol L^{-1} KO^{t}Bu

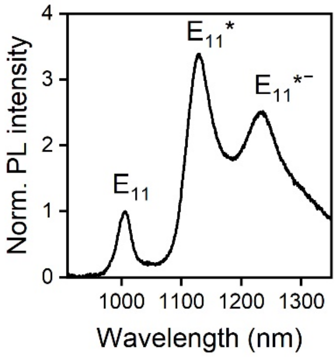

very high degree of functionalization,

higher reactivity compared to functionalization without 2-fluoroaniline (see above)

Supplementary Note 4: Non-Aniline Reagents

In contrast to functionalization with aniline derivatives, a reduced reactivity and selectivity towards $E_{11}{}^{*-}$ defects is observed for non-aniline reagents. This behaviour probably originates from the molecular differences of the carbanion intermediates. The stabilization and position of the reactive carbanion can differ vastly between different substance classes. For example, Li *et al.* showed that the reaction of C_{60}-fullerenes with indole in the presence of KO^{t}Bu/DMSO occurs via functionalization in C3-position of indole.[21] In contrast to that, phenol attacked in the C4-position.[22] For anilines the lowest relative energy of the carbanion is expected in C_2-position,[23] however, steric interaction may limit an attack in this position.
Overall the lowest energy path along the potential energy surface of the reaction can vary between different reagents and lead to significant changes in reactivity for the creation of $E_{11}{}^{*-}$ defects. When the formation of $E_{11}{}^{*-}$ is reduced, other functionalization processes (e.g., radical functionalization) may be favoured kinetically. This could lead to additional shoulders and sidebands in the PL spectrum and overall lower selectivity for one specific defect emission. This concept is supported by the very similar PL spectra obtained for reactions with 2-iodophenol and thiophenol, as they represent similar substance classes und are expected to follow similar reaction paths. Lastly, for anilines the introduction of $E_{11}{}^{*-}$ defects can be controlled through the deprotonation equilibrium and thus KO^{t}Bu concentration (see Figure 1b). This equilibrium depends on the pk_a values of the reagent.

Functionalization with Non-Aniline Reagents

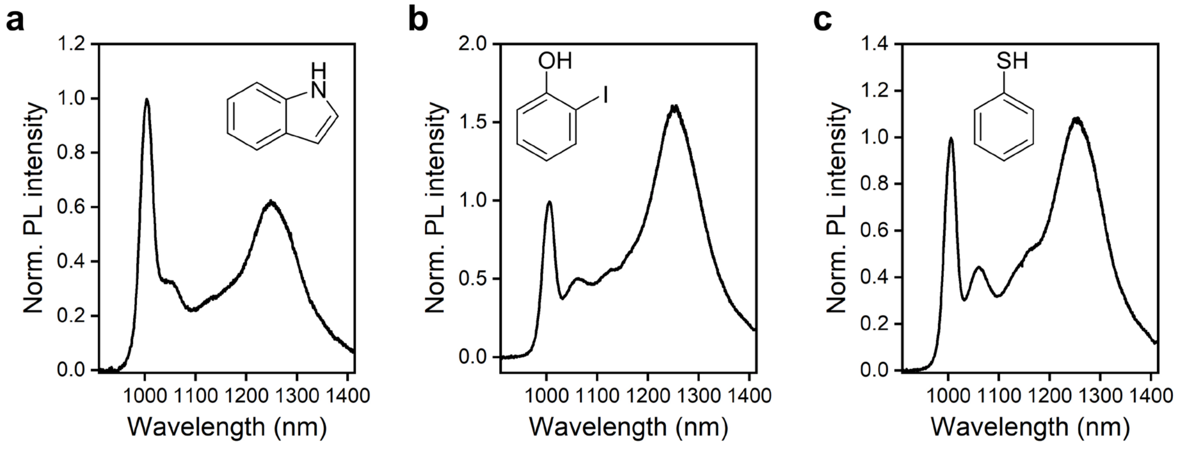

Supplementary Figure 17. a-c, Normalized PL spectra of (6,5) SWNTs functionalized in the dark with indole (**a**), 2-iodophenol (**b**) or thiophenol (**c**) and 2 eq. of KO^{t}Bu for 30 minutes in the dark in toluene/DMSO/THF. The concentration of indole, 2-iodophenol and thiophenol was kept at 29.30 mmol L^{-1}.

E_{11}*⁻ Optical Trap Depths of Functionalized (6,5) SWNTs

Supplementary Table 5. Summary of optical trap depths for $E_{11}*^{-}$ defects obtained by reaction of (6,5) SWNTs with seven different reagents. Different functional groups did not yield significant changes in optical trap depth.

Reagent	Optical trap depth, E_{11} - $E_{11}*^{-}$ (meV)
2-iodoaniline	247
2-bromoaniline	241
5-fluoro-2-iodoaniline	244
2-fluoroaniline	242
indole	244
2-iodophenol	244
thiophenol	246

Supplementary Note 5: Impact of Oxygen/Water on Functionalization

When the reaction is performed under inert conditions a strong increase in the degree of functionalization is observed. This is expected as the functionalization process may be inhibited under atmospheric conditions due to multiple effects:

(1) Quenching of the base by moisture can lead to a reduced formation of reactive carbanionic intermediates.

(2) Oxidation of negatively charged SWNT intermediates under regeneration of the carbon double bond.

(3) Oxidation of carbanionic intermediates. While the direct oxidation of the reactive carbanionic intermediate may be possible, we observed a strong dependence of the degree of functionalization on the use of DMSO as co-solvent as previously discussed. Thus, oxidation of dimsyl anions to dimethylsulfone and methanesulfonic acid is most likely.

Functionalization under Atmospheric Conditions

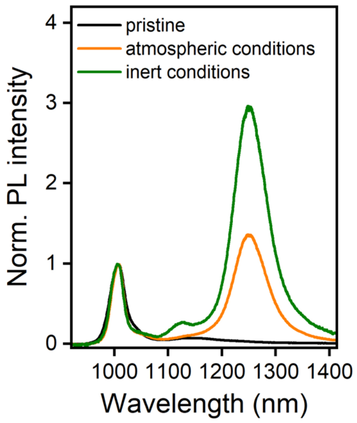

Supplementary Figure 18. Normalized PL spectra of (6,5) SWNTs functionalized in the dark with 2-iodoaniline/KO^{t}Bu under atmospheric conditions (orange) and under inert conditions (green). PFO-BPy wrapped (6,5) SWNTs in toluene were degassed by the freeze-pump-thaw method. A normalized PL spectrum of pristine (6,5) SWNTs is shown in black. The concentration of 2-iodoaniline was kept at 29.30 mmol L^{-1}.

Supplementary References

1 Berger, F. J., Lüttgens, J., Nowack, T., Kutsch, T., Lindenthal, S., Kistner, L., Müller, C. C., Bongartz, L. M., Lumsargis, V. A., Zakharko, Y. & Zaumseil, J. Brightening of Long, Polymer-Wrapped Carbon Nanotubes by *sp*^{3} Functionalization in Organic Solvents. *ACS Nano* **13**, 9259-9269 (2019).

2 Graf, A., Zakharko, Y., Schießl, S. P., Backes, C., Pfohl, M., Flavel, B. S. & Zaumseil, J. Large scale, selective dispersion of long single-walled carbon nanotubes with high photoluminescence quantum yield by shear force mixing. *Carbon* **105**, 593-599 (2016).

3 Streit, J. K., Bachilo, S. M., Ghosh, S., Lin, C. W. & Weisman, R. B. Directly measured optical absorption cross sections for structure-selected single-walled carbon nanotubes. *Nano Lett.* **14**, 1530-1536 (2014).

4 Kim, Y., Velizhanin, K. A., He, X., Sarpkaya, I., Yomogida, Y., Tanaka, T., Kataura, H., Doorn, S. K. & Htoon, H. Photoluminescence Intensity Fluctuations and Temperature-Dependent Decay Dynamics of Individual Carbon Nanotube *sp*^{3} Defects. *J. Phys. Chem. Lett.* **10**, 1423-1430 (2019).

5 Kim, M., Adamska, L., Hartmann, N. F., Kwon, H., Liu, J., Velizhanin, K. A., Piao, Y., Powell, L. R., Meany, B., Doorn, S. K., Tretiak, S. & Wang, Y. Fluorescent Carbon Nanotube Defects Manifest Substantial Vibrational Reorganization. *J. Phys. Chem. C* **120**, 11268-11276 (2016).

6 He, X., Velizhanin, K. A., Bullard, G., Bai, Y., Olivier, J. H., Hartmann, N. F., Gifford, B. J., Kilina, S., Tretiak, S., Htoon, H., Therien, M. J. & Doorn, S. K. Solvent- and Wavelength-Dependent Photoluminescence Relaxation Dynamics of Carbon Nanotube *sp*^{3} Defect States. *ACS Nano* **12**, 8060-8070 (2018).

7 Kadria-Vili, Y., Bachilo, S. M., Blackburn, J. L. & Weisman, R. B. Photoluminescence Side Band Spectroscopy of Individual Single-Walled Carbon Nanotubes. *J. Phys. Chem. C* **120**, 23898-23904 (2016).

8 Gifford, B. J., He, X., Kim, M., Kwon, H., Saha, A., Sifain, A. E., Wang, Y., Htoon, H., Kilina, S., Doorn, S. K. & Tretiak, S. Optical Effects of Divalent Functionalization of Carbon Nanotubes. *Chem. Mater.* **31**, 6950-6961 (2019).

9 Bhakuni, B. S., Kumar, A., Balkrishna, S. J., Sheikh, J. A., Konar, S. & Kumar, S. KO^{t}Bu Mediated Synthesis of Phenanthridinones and Dibenzoazepinones. *Org. Lett.* **14**, 2838-2841 (2012).

10 Sun, C. L., Li, H., Yu, D. G., Yu, M., Zhou, X., Lu, X. Y., Huang, K., Zheng, S. F., Li, B. J. & Shi, Z. J. An efficient organocatalytic method for constructing biaryls through aromatic C-H activation. *Nat. Chem.* **2**, 1044-1049 (2010).

11 Barham, J. P., Coulthard, G., Emery, K. J., Doni, E., Cumine, F., Nocera, G., John, M. P., Berlouis, L. E., McGuire, T., Tuttle, T. & Murphy, J. A. KO^{t}Bu: A Privileged Reagent for Electron Transfer Reactions? *J. Am. Chem. Soc.* **138**, 7402-7410 (2016).

12 Holzinger, M., Vostrowsky, O., Hirsch, A., Hennrich, F., Kappes, M., Weiss, R. & Jellen, F. Sidewall Functionalization of Carbon Nanotubes. *Angew. Chem. Int. Ed.* **40**, 4002-4005 (2001).

13 Olmstead, W. N., Margolin, Z. & Bordwell, F. G. Acidities of water and simple alcohols in dimethyl sulfoxide solution. *J. Org. Chem.* **45**, 3295-3299 (1980).

14 Corey, E. J. & Chaykovsky, M. Methylsulfinyl Carbanion (CH_3-SO-CH_2-). Formation and Applications to Organic Synthesis. *J. Am. Chem. Soc.* **87**, 1345-1353 (1965).

15 Buden, M. E., Bardagi, J. I., Puiatti, M. & Rossi, R. A. Initiation in Photoredox C-H Functionalization Reactions. Is Dimsyl Anion a Key Ingredient? *J. Org. Chem.* **82**, 8325-8333 (2017).

16 Bordwell, F. G. & Algrim, D. J. Acidities of anilines in dimethyl sulfoxide solution. *J. Am. Chem. Soc.* **110**, 2964-2968 (1988).

17 Graupner, R., Abraham, J., Wunderlich, D., Vencelová, A., Lauffer, P., Röhrl, J., Hundhausen, M., Ley, L. & Hirsch, A. Nucleophilic−Alkylation−Reoxidation: A Functionalization Sequence for Single-Wall Carbon Nanotubes. *J. Am. Chem. Soc.* **128**, 6683-6689 (2006).

18 Tasis, D., Tagmatarchis, N., Bianco, A. & Prato, M. Chemistry of Carbon Nanotubes. *Chem. Rev.* **106**, 1105-1136 (2006).

19 Maeda, Y., Saito, K., Akamatsu, N., Chiba, Y., Ohno, S., Okui, Y., Yamada, M., Hasegawa, T., Kako, M. & Akasaka, T. Analysis of Functionalization Degree of Single-Walled Carbon Nanotubes Having Various Substituents. *J. Am. Chem. Soc.* **134**, 18101-18108 (2012).

20 Liang, F., Sadana, A. K., Peera, A., Chattopadhyay, J., Gu, Z., Hauge, R. H. & Billups, W. E. A Convenient Route to Functionalized Carbon Nanotubes. *Nano Letters* **4**, 1257-1260 (2004).

21 Li, F., Wang, L., Wang, J., Peng, D., Zhao, Y., Li, S., Zhou, H., Wu, J., Tian, X. & Tian, Y. KO^{t}Bu-Mediated, Three-Component Coupling Reaction of Indoles, [60]Fullerene, and Haloalkanes: One-Pot, Transition-Metal-Free Synthesis of Various 1,4-(3-Indole)(organo)[60]fullerenes. *Org. Lett.* **19**, 1192-1195 (2017).

22 Li, F., Xuan, J., Zhang, S., Liu, B., Yang, J., Liu, K., Liu, D., Zhang, Q., Zhou, H., Wu, J. & Tian, Y. KO^{t}Bu-Promoted C4 Selective Coupling Reaction of Phenols and [60]Fullerene: One-Pot Synthesis of 4-[60]Fullerephenols under Transition-Metal-Free Conditions. *J. Org. Chem.* **83**, 5431-5437 (2018).

23 Vakula, N. I., Kuramshina, G. M., Makhmutova, S. F. & Pentin, Y. A. DFT theoretical studies of anions of aniline and its several derivatives. *J. Struct. Chem.* **22**, 345-356 (2010).
